# A Paradigm for Targeting Functional Impairment as an Outcome in Attention-Deficit/Hyperactivity Disorder

**DOI:** 10.3390/brainsci12081014

**Published:** 2022-07-31

**Authors:** Margaret Danielle Weiss

**Affiliations:** Cambridge Health Alliance, Harvard University, Cambridge, MA 02459, USA; margaret.weiss@icloud.com; Tel.: +1-617-665-1338

**Keywords:** functional impairment, measurement, hyperactivity, attention deficits, endpoint treatment, outcome, ADHD, remission, rating scales

## Abstract

Although functional impairment is required for a diagnosis in the DSM 5, the time frame and definition of functional impairment is ambiguous. We present a conceptual review clarifying the difference between functional impairment as a stable trait representing strength or disability in various domains, and functional impairment as secondary to emotional or behavior problems, which is a state sensitive to change with treatment intervention. Functional impairment as a measure of treatment outcome includes both change from baseline and status at the endpoint of treatment. When using a validated measure of function, functional improvement can be defined as the percentage of patients who achieve the Minimal Important Clinical Difference (MCID) and functional remission as the percentage of patients who normalize at treatment endpoint. True treatment remission should be defined as both symptomatic and functional remission.

## 1. What Is Functional Impairment? 

Psychiatrists are most comfortable working in a paradigm which frames the objective of assessment as a diagnosis, and the objective of treatment as a decrease in symptoms [1]. Patients, on the other hand, present for the most part with one or more ‘problems’. We use the clinical history, mental status, and rating scales to bridge the interface between the physician’s and the patient’s perspectives [2,3]. Since symptoms and functioning are moderately correlated [4,5], this approach has worked reasonably well. For the most part, improvement in symptoms will lead to improvement in the concern that brought the patient to treatment [6]. The focus of this article is on the relationship and timing of observable symptoms and functional domains as outcomes of improvement and remission in clinical practice. We do not address the neurobiological underpinnings of the pathogenesis of ADHD symptoms. 

In looking at response to intervention, we are most interested in the situations where symptom response and functional response are *not* consistent with one another [7]. There are patients who have a robust symptom response but are still suffering from one or another problem. For example, in a student with ADHD and a learning disability, improvement in ADHD symptoms may *not* lead to the expected academic performance without added academic remediation. When symptoms improve and the problem does not, the clinician may understand the patient to be ‘treatment resistant’, which will trigger the clinician to further probe into what might have been missed or what additional treatment is needed. The converse is also true. There are patients who may feel much improved, despite still being symptomatic. In this case, a systematic evaluation of both symptoms and functioning prompts the clinician to aggressively pursue the continued treatment of symptoms to determine if there might be a further functional improvement of latent difficulties the patient is not aware of. It is in those cases, where we see a response in either symptoms or functional impairment but not both, that we often face our most significant clinical challenges. Without systematic measurement of both symptoms and functional impairment, we will fail to identify and treat the residual difficulties essential to optimize and best practice evidence-based treatment [8]. Ultimately, true remission requires the normalization of both symptoms and function [9].

In a study evaluating the relationship between symptomatic and functional improvement in a clinical trial of a stimulant, it was found that 19% of patients who had full symptom remission were still functionally impaired and 43% of patients who had symptomatic improvement remained functionally impaired [10]. Residual functional deficits in treatment are common, impairing, and often treatable with targeted interventions to address the domain of impairment. Many targeted interventions for specific domains of functional impairment are psychosocial. Examples include cognitive behavior therapy [11,12], social skills training for difficulty with social skills, parent training for parent–child conflict [13], educational plans to address learning problems [14,15], daily report cards for school misbehavior [16], and organizational skills training [17] for difficulty with executive function. 

Unfortunately, medication management is more readily accessible than skilled evidence-based psychosocial treatment [1]. A lack of access to good psychosocial intervention for functional impairment may in part drive a lack of interest in identifying functional response as a standard of care. A comprehensive review of child treatment studies over a 15-year period between 1996 and 2011 found that 95% of studies focused on symptom outcome and less than half focused on functional impairment [18,19]. We would argue that it is function over time that predicts long-term outcome [20]. Given the wealth of data to indicate that the long-term course of ADHD is often complicated by a wide range of deleterious outcomes in adulthood [20], we should be routinely following the clinical trajectory of function over time in each patient. If we do not measure function, we will also fail to advocate for and establish the treatments that may be needed to remediate these impairments. 

In research studies, we define a ‘primary outcome’ as the most important among the many outcomes that are to be examined in the study [21]. In clinical trials in ADHD, this is almost always ADHD symptom response. ‘Secondary outcomes’ are typically exploratory and look at a wider range of effectiveness variables such as functional impairment or quality of life. I would propose that if patients, rather than clinicians, were defining the primary outcome, this might be reversed. Patients care more about whether the presenting problem has improved, than they care about whether symptoms improve as represented by a rating scale score. The patient may think: I hope he treats what I have. ‘Have’ is the problem, not the disease. The physician thinks: I hope he has what I treat. What he ‘treats’ represents is the disease, not the problem. Good treatment occurs at the intersection of both perspectives. We will conceptualize ways to adapt this paradigm to bring the patient’s and the physician’s perspectives in closer alignment. 

It is critical to distinguish *absolute* impairment as a stable patient characteristic from *relative* functional impairment driven by emotion or behavior. Absolute functional impairment may be a lifelong and relatively stable trait that characterizes the subjects’ strengths or weaknesses in comparison to population norms. Absolute function is static, making it less useful for evaluating the clinical impact of a short-term intervention. For example, someone may be intellectually delayed and unable to succeed in learning at the same rate or to the same endpoint as peers. This patient will show consistent cross-domain delays relative to peers that may continue into adulthood. A patient may be a ‘loner’, comfortable with living with an unusual degree of social isolation, although he/she does not have a psychiatric condition to explain this lack of interest in being with people. This patient has less social interest than is typical in the population, as a temperamental trait that may neither need nor respond to ‘treatment’. Understanding the absolute level of functional impairment or wellbeing is important in knowing how to structure the environment and the expectations of an individual. 

Relative functional impairment refers to functional impairment secondary to a diagnosis, symptoms, or overall emotional well-being as experienced in the normal population. By contrast with the example cited above, a child with autism may show functional impairment in social skills secondary to symptoms of autism, which may improve with specific social skills training or applied behavioral analysis. ADHD symptoms are often highly treatment responsive, in which case we will see improvement in functional impairment driven by those symptoms. For example, ADHD may lead to difficulty with classroom behavior, and when these symptoms remit, we would likely see improved functioning at school. Since relative functional impairment can be responsive to treatment and since improvement in functional impairment is the ultimate measure of the success of treatment, relative functional impairment is critical to both the clinician’s and the patient’s perspectives of treatment. 

## 2. Measurement Informed Care 

Shared decision making and evidence-based treatment depend heavily on having tools that define a common metric for evaluating symptoms and functioning [22]. These tools improve the efficiency, accuracy, and depth of assessment and are essential to best practice care. The psychometric validation of these measures means that we have immediate access to understanding severity, improvement, and whether the problem is minimally better, much better, or if the patient’s condition is no longer a concern [23]. There are two important targets for looking at response to treatment: change from baseline and outcome at the end of treatment. 

The patient’s response as measured by how they are doing at the end of the treatment is often reported by comparison with the rest of the population either as a percentile or a T score. In this paradigm, patients with an outcome score of T < 60 (1 SD) are seen as well, patients with an outcome between T = 60–65 would be seen as at risk, and patients with an outcome T > 65 (1.5 SD) would be seen as still clinically symptomatic. T scores are available on most symptom and functional measures that have population norms [24]. 

There are various ways to measure response to intervention as change from baseline to endpoint, or improvement. The ‘minimal importance difference’ or ‘minimal important change’ is defined as the level of change needed to be recognizable as meaningful by the patient or psychometrically as a half standard deviation [25] or 2–6 T score points [26]. Change is also often reported categorically either as the percentage of patients that meet a threshold % change (i.e., 35% decreased in symptoms from baseline) [27] or the percent of patients who are rated as much or very much improved as rated by the Clinical Global Impression (CGI-Improvement) [28]. 

To meaningfully describe response to treatment, it is essential to look both at change and final endpoint. Patients may show a great deal of change, and still have difficulties. Patients who present with mild symptoms or problems may show a small amount of improvement and be fully remitted at the end of treatment. Best practice treatment in ADHD requires both evidence-based treatment and measurement-informed care. This means systematically applying best practice treatment and carefully measuring baseline, outcome, and change [29]. Measurement-informed care includes the systematic assessment of both broad and syndrome-specific symptoms and broad and domain-specific functional impairments [22]. 

This facilitates communication and engagement between the patient and the doctor and becomes the cornerstone of psychoeducation [30]. A clinician providing psychoeducation shares their understanding of the relationship between symptoms and functioning with the patient. In a parent training program, for example, we may explain how oppositional symptoms may impact the parent–child relationship. Optimal treatment begins when patients and clinicians arrive at a shared explanatory framework to understand how symptoms reflect disorders that impact domains of impairment. 

## 3. Tools for Measurement Informed Assessment 

ADHD is usually comorbid [31], and a careful evaluation of this comorbidity and a differential diagnosis of the condition is an essential, and sometimes challenging, aspect of assessment. This may include broad diagnostic interviews specific to diagnosis of ADHD that include a component looking at comorbid conditions. These comprehensive interviews include past and current symptom evaluation, differential diagnosis, complemented with documentation of examples of functional impairments. The Diagnostic Interview for ADHD (DIVA) (www.divacenter.eu (accessed on 30 July 2022)) has now been translated into 25 languages and has greatly facilitated clinician acceptance and comfort with diagnostic assessment for ADHD [32,33,34,35]. DIVA-5 asks about the presence of ADHD symptoms over time, as well as lifetime functional impairments due to these symptoms. There are versions of the DIVA specific to very young children, adolescents, adults, and people with an intellectual disability. The DIVA is available for a small fee to cover costs. The ADHD Child Evaluation (ACE), or the adult version (ACE+) (https://www.psychology-services.uk.com/ACE-and-ACE-plus/ (accessed on 30 July 2022)) includes online training, interviews to guide scoring for DSM 5 or ICD-10, and specifically points to examples providing information on how each symptom impacts the patient. There are also preassessment rating scales that can be completed as a self or collateral report to guide the interview. The ACE interviews are available free of charge on the website. Diagnostic interviews are routinely used in research. However, they can also be useful clinically, especially for less experienced clinicians looking to ensure they provide a comprehensive evaluation. 

Experienced clinicians do not typically use diagnostic interviews in practice. However, even the experienced clinician will benefit from the use of a rating scale that can identify symptoms that might otherwise be missed in a quick mental status, and which also provides a quick way to get collateral regarding the relative severity of different symptom clusters. Only once the clinician has a grasp of comorbidity and differential diagnoses, should the focus turn to assessment and severity of a single condition. Unfortunately, a broad-based symptom screening is not usually done routinely. Most well validated scales in the public domain are diagnosis specific, such as the PHQ9 for depression [36] or the SCARED for anxiety [37]. Failure to use broad symptom screeners in the initial assessment may run the risk of failing to identify clinically significant comorbidity or differentials that might explain nonspecific attention or irritability difficulties. 

The most widely used broad-based rating scale is the Strengths and Difficulties Questionnaire (www.sdqinfo.org (accessed on 30 July 2022)) which is translated into 75 languages and extensively normed [38]. The DSM 5 screening tool for children (The DSM-5 Parent/Guardian-Rated Level 1 Cross-Cutting Symptom Measure—Child Age 6–17), and another for adults (DSM-5 Self-Rated Level 1 Cross-Cutting Symptom Measure—Adult), are not normed but are very useful as they follow a shared diagnostic framework [39]. These tools screen for depression, anger, irritability, mania, anxiety, somatic symptoms, inattention, suicidal ideation/attempt, psychosis, sleep disturbance, repetitive thoughts and behaviors, and substance use. The same is true for the Weiss Symptom Record–II (WSR-II) developed in Canada over the last 15 years (https://www.caddra.ca/wp-content/uploads/WSR-II.pdf (accessed on 30 July 2022)). The WSR-II groups items by diagnostic domain with a built-in scoring calculator, thus simplifying the clinicians’ ability to immediately compare and contrast the severity of different disorders. Of particular interest is the development of computerized adaptive testing (such as www.adaptivetestingtechnologies.com (accessed on 30 July 2022)) which can be understood as the personalized medicine of diagnostic assessment since this technique uses artificial intelligence to adaptively select questions based on patient responses, selecting from over 2100 items to provide an accurate assessment in less than 10 min [40]. 

Once the diagnosis of ADHD is established, and ADHD symptoms have been determined to be the objective of treatment, measurement-informed care can turn to diagnosis-specific rating scales. There are many such measures available, many of which include measurement of the 18 ADHD symptoms and 8 oppositional symptoms on a 4-point Likert scale as normal, mild, moderate, or severe with moderate and severe used to categorically define the symptom as ‘present’. By convention, on such a scale, scores of moderate or severe on six or more items of either attention or hyperactive/impulsive difficulties will be understood as meeting the criteria for ADHD symptoms. It should be emphasized that evidence of 6/9 symptoms on a rating scale for either attention or hyperactive/impulsive symptoms is necessary but not sufficient for diagnosis. The most common ADHD-specific rating scales in the public domain are the Vanderbilt (https://www.nichq.org/resource/nichq-vanderbilt-assessment-scales (accessed on 30 July 2022)) [41,42], the SNAP [43], and the ADHD-RS [44,45,46,47], which is now revised for DSM-5. 

## 4. Tools for Measurement-Informed Assessment of Functional Impairment 

The best normed measure for assessment of absolute functional impairment in ADHD is the Barkley Functional Impairment Scale (BFIS), which has been normed to measure impairment as compared to the population over the last six months [48]. This scale is available with psychometric norms for children, adolescents, and adults. The BFIS tells the clinician about disability in 15 important domains of psychosocial impairment. This would be useful in providing the patient with feedback about their strengths and weaknesses to determine appropriate expectations or needs for environmental support. 

The Weiss Functional Impairment Rating Scale is a measure of relative functional impairment which has been tested specifically in ADHD, other clinical conditions, and normative samples [49]. The Weiss Functional Impairment Rating scale (WFIRS) can be administered as a self-report for adolescents or adults (WFIRS-S), or as a parent report on their child or teen (WFIRS-P) [49]. A version of the WFIRS was also developed for collateral report in adults, such as by a spouse [49]. The focus of the WFIRS is on functional impairment, exclusive of symptoms, to the extent that this is possible. Unlike some measures of function such as the Impairment Rating Scale [50], grouping the items into specific domains allows for a multi-factorial scale that can be better used to guide intervention. Sharing a graphical display of results of the WFIRS by domain with the patient allows the clinician to help them understand the need to invest in domain-specific interventions, and then to monitor the response to those interventions over time. 

The WFIRS provides both domain-specific scores for learning and behavior at school and/or work, family, risky behavior, self-concept, and social functioning. The scales are scored by computing a mean score for each domain to allow for comparison across domains, and to ensure that items which are not applicable to a particular patient are not included in the final score. The population norms for the domains and for the total score have been derived from a study of 3130 patients (manual in preparation), with tables to determine the cut off for scores in the clinical range (T > 65), at risk (T = 60–65), or normal (T < 60). For clinical purposes, a rough and ready guide to impairment which correlates quite well with scoring based on population norms is to consider any domain with two or more items rated as moderate (2) or one item rated as severe (3). The very high internal consistency of the scale and within each domain, and the moderate correlation between domains and between each domain and the whole scale suggests that the patients perceive themselves as carrying a cross-cutting level of impairment over and above specific domain concerns. The high test/retest scores suggest that patients can also reliably report their perception of their functional impairment over time. Data from use of the WFIRS in clinical trials have clarified that the timeline to improvement in function is co-temporal with response in symptoms. Until these studies were done, it had been assumed that there would be a time lag before improvement in symptoms translated into improvement in function. This may also reflect the difference between assessment of relative vs. absolute function. Relative function may improve instantaneously, while absolute function may only change very slowly over time. 

In a recent narrative review of findings from studies of the WFIRS, psychometric validation studies conducted in a wide range of cultures and languages, using parent or self-report, and in research, clinical, or community settings showed remarkable consistency [49]. The cross-cultural stability of the WFIRS makes it ideal for comparing results internationally and for comparing response to intervention in diverse or underserved populations. 

## 5. Developmental Presentation of Functional Impairment 

The evaluation of functional impairment is critical at all stages of the life cycle, but at each developmental transition there are ‘critical’ areas that become relatively more important. While all domains are relevant at all ages, each developmental transition is associated with unique challenges which will highlight various associated critical domains. A toddler needs to acquire basic life skills such as toilet training, social play, and language. A school-aged child must develop the executive function skills to be able to learn and manage their world relatively independently. An adolescent needs to negotiate complex peer relationships and manage increasing separation from the family as an individual. A young adult must develop work skills. Failure to consolidate the acquisition of the functional skills associated with one stage of the life cycle will often impair the acquisition of the skills needed in a future stage of development. A 9-year-old who cannot get along with other children is going to have difficulty with managing independence in adolescence and avoiding high risk activities. A young adult who cannot get up on time to get to work or to school will not be able to support themself. An adult who still suffers from dangerous and impulsive behaviors such as reckless driving or substance use may as a result suffer injuries or medical morbidity that then leads to problems in the geriatric years. Clinicians need to be aware of the functional challenges specifically associated with each developmental transition to be able to interpret the impacts through the life cycle. The essence of early intervention in ADHD is to remediate those functional impairments that may complicate and impede future development. Failure to meet the demands of each developmental transition is often associated with anxiety and depression. Treatment of mood problems secondary to developmental failures requires an evaluation of the functional impairment that is leading to the developmental arrest so that the underlying concern driving the feelings of failure can be addressed. 

## 6. Targeting Functional Impairment in ADHD

ADHD is an excellent example of how a syndrome or symptom cluster drives specific functional impairments. We know, for example, that problems with attention will have a specific and clinically significant impact on academic performance, that disruptive behavior will impact peer and family relationships, or that ADHD in general will be associated with difficulties such as obesity, with driving, poor self-care, medical concerns, social isolation, high risk behaviors, substance use, marital issues, and parenting problems [51]. 

There are well described, and specific patterns associated with the impact of ADHD on particular domains, and this has informed our understanding of how to establish supportive adaptations or accommodations in the milieu. ADHD can impact academic performance due to a difficulty with poor time management, careless mistakes, dysgraphia, or lack of motivation [52,53]. Educational accommodations can be helpful in ensuring that patients with ADHD are evaluated in such a way as to ensure accurate assessment of actual academic attainment rather than performance skills such as test taking [15]. The same can be true for adult functioning in the workplace. Adults with ADHD will often self-select types of employment that are ADHD-friendly, such as information technology, sales, or any other area that is a source of committed interest for that individual [54,55,56]. Difficulty with various aspects of self-regulation such as sleep can be addressed through specific treatments including sleep hygiene [57,58,59,60]. One of the most serious areas of impairment in adults with combined type ADHD is high risk activities and given that this may be driven by relatively infrequent but salient instances of impulsivity, this can also be difficult to manage. Education of the families regarding these risks to encourage increased monitoring may be helpful. Executive dysfunction is present in many individuals with ADHD [61] and can benefit from organizational skills training [62] and the use of electronic organizational supports. The development of the International Classification of Function Core Sets for Function in ADHD [63,64,65] may help systematize the recognition of targeted functional impairments associated with ADHD and raise awareness of the need for systematic and targeted interventions [66]. 

## 7. Conclusions

We propose a paradigm shift in looking at the relationship between symptom response and function. Historically, response to intervention has been heavily weighted to improvement and remission of ‘core’ ADHD symptoms. Various conventions have been used to define improvement, such as a 30% change in symptoms, a patient being much or very much improved, or a patient demonstrating at least the defined Minimal Clinical Important Difference for that symptom measure [28]. By convention, ‘symptom remission’ has been defined as a mean score of 1 or less on the ADHD symptom scale [67]. 

This relative bias towards looking exclusively at symptom improvement and remission represents a clinician perspective on treatment. This article proposes that a more patient-centered approach to looking at outcome would include improvement or remission of the *patient’s* presenting functional complaints. Functional improvement can be defined as a 30% change score, or by using the Minimal Clinical Important Difference for a well validated functional scale. Functional remission represents an endpoint of treatment in which the patient feels ‘well’, defined on a measure with population norms as a patient rating their functional impairment as less than the ROC of a functional measure [68], or within 1 SD of the population norms for that domain or total score. 

The value of this perspective is the guidance it offers in ensuring that patients who remain impaired despite symptom improvement continue to receive the additional treatment they need [10]. A perspective that requires both symptom and functional remission as the target of intervention, it ensures that we acknowledge that both the disease and the patient must be ‘well’ for treatment to be considered a success. A further exploration of these ideas would be demonstrating that when symptoms improve, the functional impairment driven by those symptoms improves very quickly, making this more rigorous evaluation of outcome feasible even in short-term double-blind trials, without having to wait to look only at response in long-term open label. Our hope is that a routine reporting of both symptom and functional improvement and remission will eventually give us a much more nuanced appreciation of the extent to which treatment has addressed the patient’s concerns, and to identify those domains of functioning which are more sensitive or more resistant to medication treatment. 

## Data Availability

Not applicable, this paper does not report original data.
